# Understanding the Patterns of Serological Testing for COVID-19 Pre- and Post-Vaccination Rollout in Michigan

**DOI:** 10.3390/jcm10194341

**Published:** 2021-09-24

**Authors:** Zhangchen Zhao, Stephen Salerno, Xu Shi, Seunggeun Lee, Bhramar Mukherjee, Lars G. Fritsche

**Affiliations:** 1 Department of Biostatistics, University of Michigan School of Public Health, Ann Arbor, MI 48109, USA; zczhao@umich.edu (Z.Z.); salernos@umich.edu (S.S.); shixu@umich.edu (X.S.); lee7801@snu.ac.kr (S.L.); larsf@umich.edu (L.G.F.); 2 Graduate School of Data Science, Seoul National University, Seoul 08826, Korea; 3 Department of Epidemiology, University of Michigan School of Public Health, Ann Arbor, MI 48109, USA

**Keywords:** coronavirus disease 2019, serologic testing, disease prevalence, vaccination, immunity

## Abstract

Testing for SARS-CoV-2 antibodies is commonly used to determine prior COVID-19 infections and to gauge levels of infection- or vaccine-induced immunity. Michigan Medicine, a primary regional health center, provided an ideal setting to understand serologic testing patterns over time. Between 27 April 2020 and 3 May 2021, characteristics for 10,416 individuals presenting for SARS-CoV-2 antibody tests (10,932 tests in total) were collected. Relative to the COVID-19 vaccine roll-out date, 14 December 2020, the data were split into a pre- (8026 individuals) and post-vaccine launch (2587 individuals) period and contrasted with untested individuals to identify factors associated with tested individuals and seropositivity. Exploratory analysis of vaccine-mediated seropositivity was performed in 347 fully vaccinated individuals. Predictors of tested individuals included age, sex, smoking, neighborhood variables, and pre-existing conditions. Seropositivity in the pre-vaccine launch period was 9.2% and increased to 46.7% in the post-vaccine launch period. In the pre-vaccine launch period, seropositivity was significantly associated with age (10 year; OR = 0.80 (0.73, 0.89)), ever-smoker status (0.49 (0.35, 0.67)), respiratory disease (4.38 (3.13, 6.12)), circulatory disease (2.09 (1.48, 2.96)), liver disease (2.06 (1.11, 3.84)), non-Hispanic Black race/ethnicity (2.18 (1.33, 3.58)), and population density (1.10 (1.03, 1.18)). Except for the latter two, these associations remained statistically significant in the post-vaccine launch period. The positivity rate of fully vaccinated individual was 296/347(85.3% (81.0%, 88.8%)).

## 1. Introduction

Testing for the novel severe acute respiratory syndrome coronavirus 2 (SARS-CoV-2) that causes coronavirus disease 2019 (COVID-19) broadly falls into two categories: (1) reverse transcription polymerase chain reaction (RT-PCR) testing for active infection (i.e., diagnostic testing) and (2) immunoglobulin G or M (IgG or IgM) antibody testing for SARS-CoV-2 antibodies. While the former is crucial for identifying acute, likely contagious cases, the latter is important for determining prior infection status. In addition, these tests can help to estimate the duration of infection-induced antibody protection/immunity against COVID-19 and to understand the extent of community spread (seroprevalence) [1,2,3,4]. With the introduction of SARS-CoV-2 vaccines, serologic testing has naturally been used to assess vaccine-induced seropositivity [5]. 

Recent works have examined COVID-19 outcomes [6] and testing patterns [7] for the RT-PCR diagnostic test among a cohort of susceptible individuals presenting to Michigan Medicine, a primary regional health center providing COVID-19 care throughout the pandemic. These previous studies highlighted differences in diagnostic testing, infection, and hospitalization rates with respect to individual demographics, such as age, sex, race, and pre-existing comorbidities. In this paper, we further examine the pattern of serologic testing at Michigan Medicine, the characteristics of those individuals who underwent serologic testing, and the factors associated with testing positive when compared with unmatched controls.

To consider the influence of vaccinations on serology testing, we split the testing data into a pre-vaccine launch period, before 14 December 2020—the date when the first approved COVID-19 vaccines became publicly available to members of the University of Michigan Health System—and a post-vaccine launch period, after 14 December 2020. Specifically, this study seeks to: (1) describe the frequency and pattern of serologic testing at Michigan Medicine over the course of the pandemic; (2) identify characteristics associated with having an antibody test or seropositivity in the pre-vaccine launch period; (3) understand the sequences of results for both diagnostic RT-PCR and serologic testing in the pre-vaccine period; (4) identify factors associated with seropositivity in the post-vaccine launch period; and finally (5) use the Pfizer-BioNTech, Moderna, and J&J’s Janssen vaccine data to compute vaccine-induced seropositivity in terms of the rate of and time to seropositivity. As an exploratory aim, we compare seropositivity across age groups 0–18 years in pre- and post-vaccine launch period. Since vaccination was not available for these age groups by the end date of our analytic period (3 May 2021), this comparison gives us a sense of changes in infection-induced immunity in children and adolescents over this time period.

## 2. Materials and Methods

### 2.1. Study Sample

**Figure 1:**
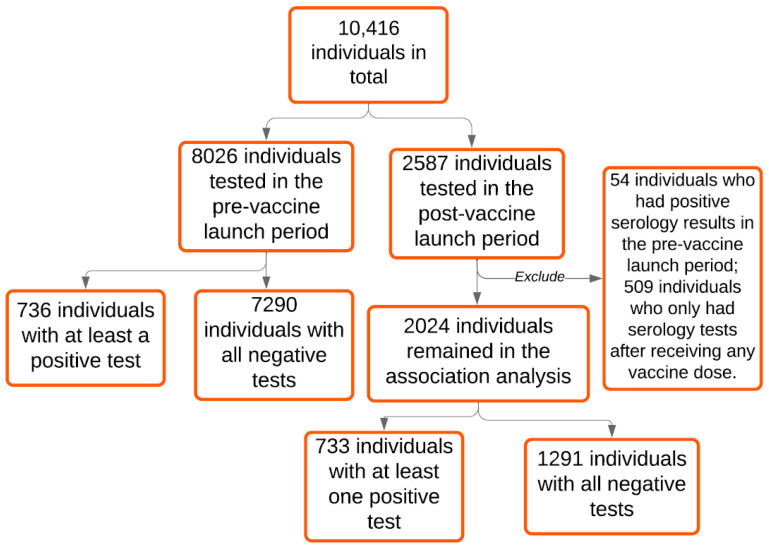
The inclusion and exclusion criteria of 10,416 individuals who had serologic tests, stratified by the date 14 December 2020.

Our study sample includes 10,416 individuals who presented to Michigan Medicine for spike protein immunoglobulin G (IgG) SARS-CoV-2 antibody testing between 27 April 2020 and 3 May 2021 (10932 tests in total). The tests’ Logical Observation Identifiers Names and Codes (LOINC) identifier was “94505-5”. Two time periods were defined: before (pre-vaccine launch) and on/after 14 December 2020 (post-vaccine launch), the date when COVID-19 vaccines roll-out began at Michigan Medicine. In the pre-vaccine launch period (27 April–14 December 2020), 8026 individuals presented for 8220 SARS-CoV-2 antibody tests, while in the post-vaccine launch period (14 December 2020–3 May 2021), 2587 individuals presented for 2712 SARS-CoV-2 antibody tests (Figure 1). For the association analysis, we excluded 54 individuals who had positive serology results in the pre-vaccine launch period to only capture novel COVID-19 infections that occurred; furthermore, because the vaccination data of unmatched controls were not available, we excluded 509 individuals who only had serology tests after receiving any vaccine dose and limited the analysis of the COVID-19 post-vaccine launch period to the individuals who had serology test results before the first vaccination dose or had no documented vaccination. Therefore, 2024 individuals remained in the post-vaccine launch period, as we wanted to tease apart factors that led to test seeking and seropositivity in the unvaccinated. 

Additionally, we utilized a cohort of unmatched controls for comparison. Unmatched controls were defined as individuals without any record of diagnostic (RT-PCR) testing or serologic testing for SARS-CoV-2 at Michigan Medicine. The control cohort was randomly selected at a case (tested individuals)-control ratio of 1:3. Individual demographic and clinical characteristics, testing rates, test results, and health outcomes were collected from the electronic medical record (EMR) on 3 May 2021. Sequences of individual-specific test results were derived from laboratory records. This cross-sectional study was approved by the committee for research ethics and compliance at Michigan Medicine and followed the Strengthening the Reporting of Observational Studies in Epidemiology (STROBE) reporting guidelines [8]. Study protocols were reviewed and determined exempt by the University of Michigan Medical School Institutional Review Board (IRB ID HUM00180294). 

### 2.2. Statistical Analysis

#### 2.2.1. Frequency and Pattern of Serologic Testing

We first described the patterns of serological testing for COVID-19 in our study population by summarizing the frequency of daily tests performed between 27 April 2020 and 3 May 2021. Pre- and post-vaccine launch periods were defined relative to the COVID-19 vaccine roll-out date, 14 December 2020. In addition, we compared the seropositivity rates across these two periods. In the COVID-19 post-vaccine launch period, we excluded individuals who had positive serology test results in the pre-vaccine launch period to only capture novel COVID-19 infections that occurred; furthermore, because the vaccination data of unmatched controls were not available, we limited the analysis of the COVID-19 post-vaccine launch period to the individuals who had serology test results before the first vaccination dose or had no documented vaccination. Specifically, we categorized tested people into three groups: children (<12 years), adolescent (≥12 and <18 years), and adults (≥18 years) because vaccination was not available for children and adolescent groups by the end date of our analytic period (3 May 2021).

#### 2.2.2. Factors Associated with Serologic Tested Individuals and Test Positivity in the Pre-Vaccine Launch Period

In our association analysis, individual characteristics included age (years), body mass index (BMI; kg/m^2^), sex (male, female, or other/unknown), race/ethnicity (non-Hispanic white, non-Hispanic black, or other/unknown), smoking status (never, current/former, or unknown), and indicators for enrolling in a COVID-19 research study led by investigators at the University of Michigan. The last variable was included, as this sub-population may have different motivation for having a test than the remaining participants. Additionally, we adjusted for seven pre-existing comorbidities extracted from the EMR: respiratory diseases, circulatory diseases, any cancers, type 2 diabetes, kidney diseases, liver diseases, and autoimmune diseases as yes/no indicators as described previously [6]. While individual-level socioeconomic status (SES) was not obtainable, we utilized three metrics of neighborhood SES based on the individual’s residence (2010 census tract) information: the proportion of the census tract population age 16+ in the civilian labor force who were unemployed (neighborhood unemployment), the proportion of the population with an annual income below the federal poverty level (neighborhood poverty), and the proportion of adults with less than a high school diploma (neighborhood education). These data were obtained from the National Neighborhood Data Archive [7] in addition to the population density of the census tract. 

We first compared individual characteristics between serology-tested individuals and unmatched controls to identify factors associated with having a serology test in the pre-vaccine launch period. Bivariate tests were conducted to determine whether the distributions of characteristics differed in these two groups, where chi-square tests and Wilcoxon rank-sum tests were used for discrete and continuous variables, respectively. In a fully adjusted logistic regression model, we regressed serologic testing outcomes (Y = 1: individuals who underwent serologic testing; Y = 0: unmatched controls) on all demographic and clinical characteristics listed above. In order to make the selection of unmatched controls more representative and less biased, when fitting the logistic regression, the set of unmatched controls were randomly selected 20 times from the patients who never underwent any COVID-19-related tests, and the case control ratio was fixed as 1:3 each time. We finally pooled 20 estimates from each logistic regression model into a single set of estimates using R package mice (version 3.13.0, R Foundation for Statistical Computing, Free Software Foundation, Boston, MA, USA). 

To identify factors associated with serology test positivity in the pre-vaccine launch period, we first compared individual characteristics between positive versus negative serology-tested individuals using a test negative design (TND). Bivariate tests were conducted to evaluate whether the distributions of characteristics differed. In order to avoid selection bias for getting tested, we further compared positive serology-tested individuals versus unmatched controls (case control-(CC)-POS) in a fully adjusted logistic regression model (Supplementary Figure S1). We regressed serologic testing outcomes (Y = 1: individuals with positive serologic test results; Y = 0: unmatched controls) on all factors repeatedly over 20 matched cohorts. The pooling technique was applied as described for the association analysis of tested individuals. 

#### 2.2.3. Patterns of Pre-Vaccine Launch Test Results for RT-PCR versus Serologic Testing 

To explore relationship between having RT-PCR tests (diagnostic) and serologic tests, we studied the sequences of testing results for each test among individuals with at least one serology test. In this analysis, only test results before vaccination (or before 14 December 2020, when the exact vaccination timing was unknown) were included to avoid effects of vaccination. 

#### 2.2.4. Factors Associated with Serological Testing in the Post-Vaccine Launch Period

Between 14 December 2020 and 3 May 2021, a large fraction of the serologic testing was likely performed to confirm antibody response to the vaccine. Therefore, we first presented descriptive statistics for all individuals with at least a serologic test and unmatched controls to identify factors associated with having a serology test in the post-vaccine launch period. The adjusted association analyses with unmatched controls were further carried out to identify factors associated with tested individuals in the post-vaccine launch period as described for the analysis of the dataset from the pre-vaccine launch period. 

To identify factors associated with serology test positivity in the post-vaccine launch period, we first compared individual characteristics between positive versus negative serology-tested individuals using a test negative design (TND). The adjusted association analyses using CC-POS were then carried out to identify factors associated with serology test positivity in the post-vaccine launch period, consistent with the analysis from the pre-vaccine launch period. 

#### 2.2.5. Vaccination Timing and Estimation of Seropositivity

We used the available vaccination data on Pfizer-BioNTech, Moderna, and J&J/Janssen vaccines, where the first two vaccines required a priming dose, followed by a booster dose for most protection, while the third is a single-dose vaccine. We reviewed the adherence to the recommended vaccination schedule of the CDC. Furthermore, we determined serology positivity rates of individuals vaccinated with Pfizer-BioNTech, Moderna, or J&J/Janssen who never tested positive before vaccination to estimate the vaccine-mediated immunity at different time points (2–4, 4–8, 8–12, or >12 weeks after the second dose). Finally, to have a rough idea of the length of time to develop a positive antibody response after vaccination, we calculated the distributions of time to first positive serologic test after an individual’s first and second doses, respectively.

## 3. Results

### 3.1. Frequency and Pattern of Serologic Testing

**Figure 2:**
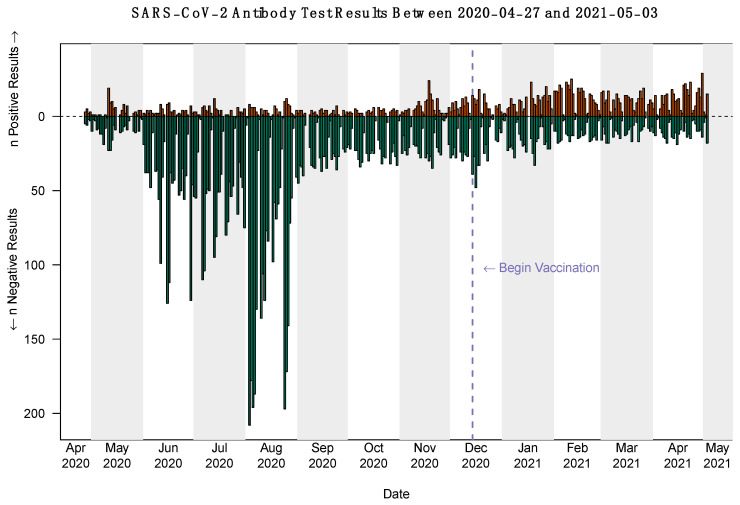
Serology testing results at Michigan Medicine between 27 April 2020 and 3 May 2021.

Between 27 April 2020 and 3 May 2021, 10,416 individuals presented to Michigan Medicine for at least a SARS-CoV-2 antibody test (Figure 2; 10,932 tests in total). Specifically, in the pre-vaccine launch period (27 April–14 December 2020), 8026 individuals presented for 8220 SARS-CoV-2 antibody tests, while in the post-vaccine launch period (14 December 2020–3 May 2021), 2587 individuals presented for 2712 SARS-CoV-2 antibody tests (Table 1a and Supplementary Figure S2). As shown, a higher proportion of adults had serology tests in the pre-vaccine launch period (7755 individuals (96.6%)) as compared to post-vaccine launch period (2394 individuals (92.5%)).

Serology testing peaked in the summer of 2020, with 84.6 daily tests on average in August. After August, the number of daily tests decreased greatly (Supplementary Table S1). Further, the seropositivity before 14 December 2020 was 9.2%, and this rate increased to 46.7% afterwards. A part of the difference was driven by the fact that 509 individuals only had serology tests after vaccination. After removing these 509 individuals as well as 54 individuals who tested positive before 14 December, the overall seropositivity after 14 December was 36.2% (Table 1b). The seropositivity of children under age 12 was 8.6% before 14 December and 26.2% afterwards. For adolescents (age ≥ 12 and <18), the seropositivity was 13.0% before 14 December and 29.4% afterwards. Since COVID-19 vaccines were not approved for these two groups at the time of our study, the observed increase of seropositivity in the second period was not the result of undocumented vaccine-mediated immunity but possibly due to the wide spread of the second wave of COVID-19 and silent asymptomatic infections.

### 3.2. Factors Associated with Serologically Tested Individuals and Test Positivity in the Pre-Vaccine Launch Period

To identify factors associated with serologically tested individuals in the pre-vaccine launch period, we compared patient characteristics between serology tested and random, untested control individuals. Each of the patient characteristics was marginally associated with tested individuals (*p*-value < 0.05 in bivariate tests of association) and each of them except for BMI and pre-existing type 2 diabetes and kidney diseases remained significantly associated when adjusting for all other variables. For example, female sex, higher population density, and indictors of pre-existing conditions (respiratory diseases, circulatory diseases, any cancer, liver diseases, and autoimmune diseases) were associated with an increased probability of having a serologic test, while age, ever-smoking status, and neighborhood SES variables (unemployment, poverty, and education rates) were associated with a decreased probability of having a serologic test (Supplementary Table S2). 

Table 2 directly compares individuals with all negative serology results and at least one positive test result in the pre-vaccine launch period. Age, sex, race/ethnicity, smoking status, neighborhood SES variables, population density, pre-existing circulatory diseases and kidney diseases, and enrollment in a COVID-19 research study were marginally associated with tested individuals (*p*-value < 0.05 in bivariate tests of association). However, some of the associations were in the reverse direction than expected, questioning the potential bias due to tested individuals affecting both positive and negative groups. For example, circulatory diseases were more common in individuals with all negative tests (66%) than in individuals with at least one positive test result (62%). 

To account for selection bias and determine patient characteristics that are associated with the risk of a past COVID-19 infection, we fit the fully adjusted logistic regression model in the CC-POS design. We found seven characteristics that are associated in the CC-POS analysis (age, non-Hispanic Black, ever-smoker, population density, respiratory disease, circulatory disease, and liver disease). Specifically, age (10 year; CC-POS OR 0.80 (0.73, 0.89)) and ever-smoker (0.49 (0.35, 0.67)) had protective effects on serology test positivity, while non-Hispanic Black (2.18 (1.33, 3.58)), population density (1.10 (1.03, 1.18)), respiratory disease (4.38 (3.13, 6.12)), circulatory disease (2.09 (1.48, 2.96)), and liver disease (2.06 (1.11, 3.84)) increased the odds for testing positive (Table 2 and Supplementary Figure S3). 

### 3.3. Factors Associated with Serologic Tested Individuals and Test Positivity in the Post-Vaccine Launch Period

In the post-vaccine launch period, there were 2587 individuals with at least one serology test. Among them, 1456 (56%) received at least one vaccine dose, and 953 (37%) were fully vaccinated (Table 1b), suggesting getting vaccinated as a significant factor for having a serologic test.

To only capture novel COVID-19 infections that occurred in the COVID-19 post-vaccine launch period, we excluded 54 individuals who had positive serology test results in the pre-vaccine launch period; furthermore, we limited the analysis of the COVID-19 post-vaccine launch period to a total of 2024 individuals who had obtained serology test results before the first vaccination dose (911 individuals) or had no documented vaccination (1113 individuals). 

To identify other factors except getting vaccinated that are associated with serologic tested individuals in the 2024 individuals in the post-vaccine launch period (Table 1b), we compared patient characteristics between these serology tested individuals and random, untested control individuals. All the patient characteristics except population density were nominally associated with tested individuals (*p*-value < 0.05 in bivariate tests of association). When adjusting for other variables, age, sex, smoking status, neighborhood SES variables (unemployment and education rates), and pre-existing conditions (respiratory diseases, circulatory diseases, any cancer, liver diseases, and autoimmune diseases) remained significantly associated (Supplementary Table S3). These association results were consistent to the serologic tested individuals’ pattern in the pre-vaccine launch period. 

In addition, Table 3 compares individuals with at least a serologic test stratified by the serologic results in the post-vaccine launch period. Smoking status, neighborhood unemployment, receiving at least one vaccine dose, being fully vaccinated, and being enrolled in a COVID-19 research study were marginally associated with seropositivity (*p*-value < 0.05 in bivariate tests of association). When determining patient characteristics that are associated with seropositivity in the post-vaccine launch period using a fully adjusted logistic regression model, we observed significant differences in age, smoking status, neighborhood education, respiratory disease, circulatory disease, kidney disease, liver disease, and autoimmune disease (Table 3 and Supplementary Figure S4). In particular, age (10 year; CC-POS OR 0.82 (0.75, 0.91)) and ever-smoker (0.70 (0.52, 0.96)) had protective effects on serology test positivity, while respiratory disease (3.09 (2.24, 4.26)), circulatory disease (2.02 (1.44, 2.84)), liver disease (2.05 (1.08, 3.89)), and autoimmune disease (2.53 (1.61, 3.96)) increased the odds for testing positive. Among them, age, smoking status, respiratory disease, circulatory disease, and liver disease were previously identified in the analysis of the pre-vaccine launch cohort.

### 3.4. Patterns of Pre-Vaccine Launch Test Results for RT-PCR versus Serologic Testing 

To understand the patterns of diagnostic versus serologic testing before vaccination, we added available RT-PCR test data for the 8732 individuals with at least one serologic test before vaccination (Supplementary Table S4) and explored their combinations and temporal sequence. There were 3649 individuals who had at least one serologic test following a diagnostic test. Among these, 3109 (85.2%) had consistent diagnostic and serologic tests: 2587 (70.9%) tested negative in both, while 522 (14.3%) with a positive diagnostic test subsequently had a positive serologic test. A total of 371 individuals (10.2%) with a positive diagnostic test subsequently only tested negative, while 169 individuals (4.6%) with a negative diagnostic had a positive serologic result afterwards (Table 4). The latter might represent potentially asymptomatic COVID-19 cases.

The numbers of individuals who had a positive and a subsequent negative serologic test was four, with 5, 21, 48, and 76 days between tests, respectively. However, since no preceding diagnostic test was available, an estimation about the immunity after a COVID-19 infection was not possible. Among the 528 individuals who had a positive diagnostic and a subsequent positive serologic test, the maximal time span between the first positive diagnostic and the last positive serologic test was 368 days, with no known new infection in between, indicating that immunity after a COVID-19 infection might last up to a year in certain individuals.

### 3.5. Estimating the COVID-19 Vaccine-Mediated Immunity 

Among the 10,416 individuals for which we had serology test results, 4653 individuals had, at the time of our study, received both doses of either the Pfizer/BioNTech or Moderna COVID-19 vaccine. We used their vaccination data to summarize the time between their first and second doses (Table 5 and Supplementary Figure S5). Most individuals (4635 individuals, 99.6%) were vaccinated within the allowable time frame. Further, 537 individuals who received their first dose and had at least 42 days follow-up time did not receive their second dose at the time of the study.

Table 6 further compares positivity rates among 139 individuals who had serology tests between doses and 347 individuals who had serology tests after being fully vaccinated, i.e., were tested at least two weeks after two doses of Pfizer-BioNTech or Moderna vaccines or the single dose of Janssen vaccine. Individuals who tested positive before vaccination were excluded. After the first and before the second dose, the positivity rate of individuals who never tested positive before was 56.1% (78/139) 95% CI (47.5%, 64.4%). This positivity rate was 85.3 (296/347) (81.0%, 88.8%) after being fully vaccinated, demonstrating adequate vaccine-mediated immunity. The positivity rate of Pfizer-BioNTech (86.9% (82.2%, 90.6%)) was slightly higher than Moderna (80.0% (67.9%, 88.5%)) and J&J/Janssen (78.6% (48.8%, 94.3%)). In general, we observed an increasing positivity rate for all three vaccines over the three months following full vaccination status (Table 6). We also estimate the length of time necessary to develop a positive antibody response after vaccination: the median time to first positive serologic result was 21 days (1st quantile: 16 days; mean: 25 days; 3rd quantile: 31 days) after the first dose and 27.0 days (1st quantile: 17 days; mean: 34 days; 3rd quantile: 45 days) after the second dose (Supplementary Figure S6).

## 4. Discussion

This study sought to quantify patterns of serologic testing for COVID-19 and its associated individual-level factors. Serological tests are clinically and epidemiologically relevant, as they can provide insights into the extent of past infection in the community and, when repeated, can inform us regarding waning immunity after infection and/or vaccination. We recognize that serology test results from EHRs are likely biased because people who undergo serological testing might not be representative of the general population. Furthermore, test-seeking behavior might change over time and thus further bias downstream analyses. For example, with the availability of COVID-19 vaccines, there was an increased interest in confirming and tracking vaccine-mediated immunity. Understanding factors that influence test-seeking behavior is thus critical to appreciate biases and their changes over time and consequently to enable statistical approaches to counteract them when estimating community seroprevalence (e.g., through inverse probability weighting). The combination of diagnostic RT-PCR results and antibody test results can lend insight into asymptomatic individuals who never tested positive for active infection but happen to be seropositive. Seroconversion is of interest for many such reasons.

In both pre-vaccine and post-vaccine launch period, we found that age, ever-smoking status, and neighborhood SES (unemployment, and education rates) were consistently associated with a decreased probability of having a serologic test, while female sex, higher population density, and indictors of pre-existing conditions (respiratory, circulatory, cancer, and liver and autoimmune diseases) were consistently associated with an increased probability of having a serologic test. 

Seropositivity in the pre-vaccine launch period was 9.2% and increased to 36.2% (after removing tests after vaccination) in the post-vaccine launch period, an increasing trend that was observable in adults (9.1 to 37.1%) but also noted to some extent in adolescents (13.0 to 29.4%) and children (8.6 to 26.2%). The latter two age groups were not eligible for vaccination at the time of this analysis. Earlier studies claims that children made up a small percentage of individuals with COVID-19 [9] and that the majority had social interactions with peers or parents rather than with older people who were at risk of severe diseases [10]. According to our study, both children and adolescents were also affected from the wide spread of the second wave of COVID-19. 

In addition, we compared the observed seropositivity with the cumulative incidence of COVID-19 at proximal timeframe. In the pre-vaccine launch period, the number of accumulated reported COVID-19 cases before 14 December 2020 was 0.547 million in Michigan (estimated population of 9.99 million [11]) and 12,795 in Washtenaw County (the location of Michigan Medicine; estimated population of 363,837 [11]) [12]. However, the detected case number likely only reflects part of the true COVID-19 cases. According to CDC, the estimated under-report factor was 4.2 (95% CI (3.6, 4.9)) [13]. Therefore, in this period, the adjusted disease prevalence was estimated to be 23.0% (19.7%, 26.8%) in Michigan and 14.8% (12.7%, 17.2%) in Washtenaw County. In the post-vaccine launch period, the number of accumulated cases of COVID-19 before 3 May 2021 (the end date of our study) was 0.970 million in Michigan and 25,952 in Washtenaw County [12]. In this period, we thus estimated the adjusted disease prevalence to be 40.8% (35.0%, 47.6%) in Michigan and 30.0% (25.7%, 35.0%) in Washtenaw County. In addition, during this period, COVID-19 vaccines were available and administered; a total of 4.32 million (43.2%) individuals in Michigan [14] and 187,800 (51.6%) individuals in Washtenaw County [15] received at least one vaccine dose. Combing the adjusted infection and vaccination data together, the serology test positivity rate in the period was estimated to be 43.2~84.0% in Michigan and 51.6~81.6% in Washtenaw County. However, both estimated serology test positivity rates were quite different from the estimated prevalence (9.2% and 36.2% for the pre- and post-vaccine launch periods) in our serologic data. There were three main explanations: (1) the under-report factor estimated from CDC might not be accurate for Michigan [16,17,18]; (2) most people received vaccination in the last two months of our study, but the number of serology tests in these two months was limited in our study; and (3) according to our association analysis, people who had serologic tests were more likely to have prior medical issues compared to the general population. Therefore, the estimated prevalence in our study might not be representative for Washtenaw County or the State of Michigan. 

We further compared differences in factors associated with seropositivity in the CC-POS analysis and found five consistent factors in both pre- and post-vaccine launch periods: age and ever-smoking had a decreased odd for testing positive, while pre-existing respiratory, circulatory, and liver diseases increased the odds for testing positive. Particularly, ever-smoking status was associated with both getting serologic tests and receiving negative serologic results when tested. This finding might support an earlier study [19] that claimed that the risk of infection by COVID-19 was reduced by half among current smokers. In addition, pre-existing conditions (respiratory disease, circulatory disease, and liver disease) were associated with serologic tested individuals and receiving positive serologic results when tested, a finding supported by earlier studies [20,21]. Both race/ethnicity and population density were significantly associated with seropositivity only in the pre-vaccine launch period. 

Similar results are noted with respect to individual sex and comorbidity burden in an early study of IgM-IgG antibody testing for COVID-19 [22]. Differences in diagnostic RT-PCR testing rates and positivity across these individual-level characteristics have also been studied previously, suggesting that similar factors for symptomatic infection may persist as predictors of later seroconversion [6,7,23]. These results shed light on the importance of population-wide serologic testing to inform on the prevalence of the disease, as those tested represent only a selected subset of the general population. 

Our study also found that 528 individuals with an initial positive diagnostic test later tested positive for IgG antibodies before being vaccinated, with a median of 45 days (max: 368 days) between the first positive diagnostic test and last positive serologic test. This immunity results were consistent with previous findings [24,25]. However, these results cannot yet shed light on long-term immunity [26]. Further, as there is evidence of reinfection among recovered individuals, Refs. [27,28] it is recommended that convalescent COVID-19 individuals receive the vaccination [29]. 

Another aim of this study was to examine patterns of serologic testing results in a large cohort of individuals receiving the Pfizer-BioNTech, Moderna, or J&J’s Janssen vaccines. Results from early studies on the vaccines showed that serum IgG concentrations and SARS-CoV-2 neutralizing titers increased notably fourteen days after an individual’s second dose [30,31]. Our study observed only fourteen individuals (16.5%) with negative serologic results beyond two weeks after receiving the first dose. A longer period of follow-up as well as aggregated data from a diverse set of regional testing centers is necessary to validate these findings beyond the Michigan Medicine specific data in our study’s time frame. 

Additionally, we note that there are prevailing limitations in using electronic health records for research due to differing purposes for data collection and use. In particular, we cannot confirm whether the patients with no vaccination record were vaccinated elsewhere. Despite these limitations, this report presents a holistic description of serologic testing patterns and results at an academic medical center near a severely affected city (Detroit, MI) during the pandemic over one year after its onset. Factors associated with seropositivity in the pre- and post-vaccine launch cohorts included age, smoking status, respiratory disease, circulatory disease, and liver disease. The majority of fully vaccinated individuals in this study had developed antibody protection against COVID-19 after two weeks of their final dose. 

## Figures and Tables

**Table 1:** (**a**). Characteristics of 10,416 individuals who had at least one serologic test, stratified by Test Period. (**b**). Test positivity rate of 9947 individuals who had at least one serologic test before vaccination, stratified by Test Period.

	Overall	Test Period	
		Before 14 December 2020 *	On/After 14 December 2020
Number of Individuals	10,416	8026	2587
Mean Age at First Test (S.D.)	43.41 (18.78)	43.28 (18.29)	43.71 (20.21)
Age Category in Years (%)			
Below 12	240 (2.3)	140 (1.7)	106 (4.1)
Adolescent (>12 and <18)	215 (2.1)	131 (1.6)	87 (3.4)
Adult (≥18)	9961 (95.6)	7755 (96.6)	2394 (92.5)
Female (%)	6388 (61.3)	4926 (61.4)	1598 (61.8)
Race/Ethnicity (%)			
Non-Hispanic White	7941 (76.2)	6116 (76.2)	1987 (76.8)
Non-Hispanic Black	663 (6.4)	543 (6.8)	133 (5.1)
Other/Unknown	1812 (17.4)	1367 (17.0)	467 (18.1)
COVID-19 Research Study Participation			
Yes	2128 (20.4)	1955 (24.4)	200 (7.7)
No	8288 (79.6)	6071 (75.6)	2387 (92.3)
COVID-19 Test Result **			
Before 1. Vaccination Dose			
Positive	1017 (11.5)	736 (9.2)	281 (30.8)
Negative	7864 (88.5)	7290 (90.8)	630 (69.2)
After 1. Before 2. Vaccination Dose			
Positive	n/a	n/a	46 (50.0)
Negative			46 (50.0)
After 2. Vaccination Dose			
Positive	n/a	n/a	337 (86.7)
Negative			52 (13.3)
Unknown Vaccination Status			
Positive	n/a	n/a	455 (40.9)
Negative			658 (59.1)
* Assuming no vaccinations before 14 December 2020. ** Some individuals were tested multiple times at various time points.			
	**Tested Positive/Tested (%)**		
	**Overall**	**Test Period**	
		Before 14 December 2020 *	On/After 14 December 2020 **
Overall	1469/9947 (14.8)	736/8026 (9.2)	733/2024 (36.2)
Age Category in Years			
Below 12	39/240 (16.2)	12/140 (8.6)	27/103 (26.2)
Adolescent (>12 and <18)	42/214 (19.6)	17/131 (13.0)	25/85 (29.4)
Adult (≥18)	1388/9493 (14.6)	707/7755 (9.1)	681/1836 (37.1)
Sex			
Female	855/6073 (14.1)	420/4926 (8.5)	435/1217 (35.7)
Male	614/3874 (15.8)	316/3100 (10.2)	298/807 (36.9)
Race/Ethnicity			
Non-Hispanic White	1051/7570 (13.9)	497/6116 (8.1)	554/1541 (36.0)
Non-Hispanic Black	142/648 (21.9)	104/543 (19.2)	38/113 (33.6)
Other/Unknown	276/1729 (16.0)	135/1367 (9.9)	141/370 (38.1)
COVID-19 Research Study Participation			
Yes	95/2046 (4.6)	67/1955 (3.4)	28/104 (26.9)
No	1374/7901 (17.4)	669/6071 (11.0)	705/1920 (36.7)
* Assuming no vaccinations before 14 December 2020. ** Excluding 54 individuals who tested positive before 14 December and 509 individuals who were only tested after vaccination; excluding results from tests after vaccination.			

**Table 2:** Characteristics of 8026 individuals with at least a serologic test before 14 December 2020, stratified by the serologic results. Statistics presented are median (inter-quartile range) for continuous variables and *n* (%) for categorical variables. Unadjusted *p*-values are reported for either Wilcoxon rank-sum (continuous) or chi-square tests of independence (categorical) comparing the distributions of each of these characteristics between testing groups. Odds ratios and 95% confidence intervals are reported for each characteristic, fully adjusting for all other demographic and clinical characteristics in a logistic regression model. When fitting the logistic regression, Y = 1 for individuals with positive serologic results; Y = 0 for untested controls. The controls were randomly selected 20 times, and the case control ratio was fixed as 1:3 each time. We finally pooled 20 estimates from each model into a single set of estimates.

	Unadjusted Comparisons				Adjusted Comparisons		
Characteristic	Overall, *n* = 8026 ^1^	Individuals with All Negative Serologic Results, *n* = 7290 ^1^	Individuals with at Least One Positive Serologic Result, *n* = 736 ^1^	*p*-Value ^2^	OR ^3^	95% CI ^3^	*p*-Value
Age, per 10 years	4.4 (2.8, 5.9)	4.4 (2.9, 5.9)	4.0 (2.2, 5.5)	**<0.001**	0.80	0.73, 0.89	**<0.001**
Body Mass Index	27 (23, 31)	27 (23, 31)	26 (23, 31)	>0.9	1.00	0.98, 1.03	0.668
Sex				**0.012**			
Male	3100 (39%)	2784 (38%)	316 (43%)		—	—	
Female	4926 (61%)	4506 (62%)	420 (57%)		1.31	0.99, 1.74	0.061
Race/Ethnicity				**<0.001**			
Non-Hispanic White	6116 (76%)	5619 (77%)	497 (68%)		—	—	
Non-Hispanic Black	543 (6.8%)	439 (6.0%)	104 (14%)		2.18	1.33, 3.58	**0.003**
Other/Unknown	1367 (17%)	1232 (17%)	135 (18%)		1.04	0.7, 1.54	0.855
Smoking Status				**<0.001**			
Never	1898 (24%)	1774 (24%)	124 (17%)		—	—	
Current/Former	5427 (68%)	4882 (67%)	545 (74%)		0.49	0.35, 0.67	**<0.001**
Unknown	701 (8.7%)	634 (8.7%)	67 (9.1%)		0.42	0.18, 1	0.052
Neighborhood Unemployment ^4^	5.10 (3.76, 7.09)	5.01 (3.76, 7.03)	5.64 (4.02, 8.15)	**<0.001**	0.98	0.92, 1.04	0.533
Neighborhood Poverty ^4^	6 (4, 12)	6 (4, 12)	7 (4, 17)	**0.001**	0.98	0.97, 1	0.052
Neighborhood Education ^4^	4.7 (2.7, 8.2)	4.6 (2.7, 8.0)	5.5 (3.1, 9.3)	**<0.001**	0.97	0.94, 1	0.085
Population Density, 1000 persons per square mile	1.84 (0.50, 3.43)	1.79 (0.48, 3.38)	2.46 (0.86, 3.86)	**<0.001**	1.10	1.03, 1.18	**0.007**
Respiratory Diseases				0.14			
No	1450 (20%)	1333 (20%)	117 (18%)		—	—	
Yes	5920 (80%)	5369 (80%)	551 (82%)		4.38	3.13, 6.12	**<0.001**
Circulatory Diseases				**0.014**			
No	2518 (34%)	2261 (34%)	257 (38%)		—	—	
Yes	4852 (66%)	4441 (66%)	411 (62%)		2.09	1.48, 2.96	**<0.001**
Any Cancer				0.4			
No	5673 (77%)	5150 (77%)	523 (78%)		—	—	
Yes	1697 (23%)	1552 (23%)	145 (22%)		1.20	0.83, 1.72	0.328
Type 2 Diabetes				0.2			
No	6499 (88%)	5921 (88%)	578 (87%)		—	—	
Yes	871 (12%)	781 (12%)	90 (13%)		1.25	0.79, 1.98	0.349
Kidney Diseases				**<0.001**			
No	6811 (92%)	6219 (93%)	592 (89%)		—	—	
Yes	559 (7.6%)	483 (7.2%)	76 (11%)		1.56	0.92, 2.64	0.1
Liver Diseases				>0.9			
No	6776 (92%)	6161 (92%)	615 (92%)		—	—	
Yes	594 (8.1%)	541 (8.1%)	53 (7.9%)		2.06	1.11, 3.84	**0.024**
Autoimmune Diseases				0.6			
No	6122 (83%)	5562 (83%)	560 (84%)		—	—	
Yes	1248 (17%)	1140 (17%)	108 (16%)		1.33	0.84,2.12	0.227
Enrolled in a COVID-19 Research study				**<0.001**			
No	6071 (76%)	5402 (74%)	669 (91%)		—	—	
Yes	1955 (24%)	1888 (26%)	67 (9.1%)		—	—	

^1^ Statistics presented: median (IQR); *n* (%). ^2^ Statistical tests performed: Wilcoxon rank-sum test; chi-square test of independence; Fisher’s exact test. ^3^ OR, odds ratio; CI, confidence interval. ^4^ The unit of neighborhood unemployment is 1% proportion of population aged 16+ in the civilian labor force who are unemployed; the unit of neighborhood poverty is 1% proportion of population with annual income below the federal poverty level; and the unit of neighborhood education is 1% proportion of adults with less than high school diploma in 2010. The bold are indicate the significance.

**Table 3:** Characteristics of 2587 individuals with at least a serologic test on/ after 14 December 2020, stratified by the serologic results. Statistics presented are median (inter-quartile range) for continuous variables and *n* (%) for categorical variables. Unadjusted *p*-values are reported for either Wilcoxon rank-sum (continuous) or chi-square tests of independence (categorical) comparing the distributions of each of these characteristics between testing groups. Odds ratios and 95% confidence intervals are reported for each characteristic, fully adjusting for all other demographic and clinical characteristics in a logistic regression model. When fitting the logistic regression, we excluded 54 individuals who had positive serology results in the pre-vaccine launch period and excluded 509 individuals who only had serology tests after receiving any vaccine dose. Y = 1 for individuals with positive serologic results; Y = 0 for untested controls. The controls were randomly selected 20 times, and the case control ratio was fixed as 1:3 each time. We finally pooled 20 estimates from each model into a single set of estimates.

	Unadjusted Comparisons				Adjusted Comparisons		
Characteristic	Overall, *n* = 2587 ^1^	Individuals with all Negative Serologic Results, *n* = 1379 ^1^	Individuals with at Least One Positive Serologic Result, *n* = 1208 ^1^	*p*-Value ^2^	OR ^3^	95% CI ^3^	*p*-Value
Age, per 10 years	4.6 (2.4, 6.1)	4.5 (2.6, 6.0)	4.6 (2.2, 6.1)	0.5	0.82	0.75, 0.91	**<0.001**
Body Mass Index	27 (23, 31)	27 (23, 31)	27 (23, 31)	0.4	1.00	0.98, 1.03	0.678
Sex				0.2			
Male	989 (38%)	542 (39%)	447 (37%)		—	—	
Female	1598 (62%)	837 (61%)	761 (63%)		1.20	0.90, 1.61	0.215
Race/Ethnicity				0.14			
Non-Hispanic White	1987 (77%)	1059 (77%)	928 (77%)		—	—	
Non-Hispanic Black	133 (5.1%)	81 (5.9%)	52 (4.3%)		0.92	0.5, 1.69	0.787
Other/Unknown	467 (18%)	239 (17%)	228 (19%)		0.88	0.59, 1.31	0.526
Smoking Status				**0.003**			
Never	663 (26%)	391 (28%)	272 (23%)		—	—	
Current/Former	1762 (68%)	908 (66%)	854 (71%)		0.70	0.52, 0.96	**0.025**
Unknown	162 (6.3%)	80 (5.8%)	82 (6.8%)		0.22	0.07, 0.71	**0.012**
Neighborhood Unemployment ^4^	5.25 (3.84, 7.19)	5.22 (3.80, 7.00)	5.34 (3.90, 7.45)	**0.044**	1.00	0.94, 1.06	1
Neighborhood Poverty ^4^	27 (23, 31)	27 (23, 31)	27 (23, 31)	0.4	0.98	0.96, 1	0.06
Neighborhood Education ^4^				0.2	0.96	0.93, 0.99	**0.018**
Population Density, 1000 persons per square mile	989 (38%)	542 (39%)	447 (37%)		1.02	0.95, 1.1	0.599
Respiratory Diseases	1598 (62%)	837 (61%)	761 (63%)				
No				0.14	—	—	
Yes	1987 (77%)	1059 (77%)	928 (77%)		3.09	2.24, 4.26	**<0.001**
Circulatory Diseases	133 (5.1%)	81 (5.9%)	52 (4.3%)				
No	467 (18%)	239 (17%)	228 (19%)		—	—	
Yes				**0.003**	2.02	1.44, 2.84	**<0.001**
Any Cancer	663 (26%)	391 (28%)	272 (23%)				
No	1762 (68%)	908 (66%)	854 (71%)		—	—	
Yes	162 (6.3%)	80 (5.8%)	82 (6.8%)		1.21	0.85, 1.72	0.288
Type 2 Diabetes				0.4			
No	2082 (87%)	1113 (86%)	969 (88%)		—	—	
Yes	314 (13%)	176 (14%)	138 (12%)		1.23	0.78, 1.94	0.383
Kidney Diseases				0.090			
No	2107 (88%)	1147 (89%)	960 (87%)		—	—	
Yes	289 (12%)	142 (11%)	147 (13%)		1.64	0.98, 2.73	0.059
Liver Diseases				0.3			
No	2186 (91%)	1183 (92%)	1003 (91%)		—	—	
Yes	210 (8.8%)	106 (8.2%)	104 (9.4%)		2.05	1.08, 3.89	**0.029**
Autoimmune Diseases				0.4			
No	1882 (79%)	1004 (78%)	878 (79%)		—	—	
Yes	514 (21%)	285 (22%)	229 (21%)		2.53	1.61, 3.96	**<0.001**
Received at least one vaccine dose				**<0.001**			
No	1131 (44%)	661 (48%)	470 (39%)		—	—	
Yes	1456 (56%)	718 (52%)	738 (61%)		—	—	
Fully vaccinated				**<0.001**			
No	1634 (63%)	948 (69%)	686 (57%)		—	—	
Yes	953 (37%)	431 (31%)	522 (43%)		—	—	
Enrolled in a COVID-19 research study				**0.002**			
No	2387 (92%)	1293 (94%)	1094 (91%)		—	—	
Yes	200 (7.7%)	86 (6.2%)	114 (9.4%)		—	—	

^1^ Statistics presented: median (IQR); *n* (%). ^2^ Statistical tests performed: Wilcoxon rank-sum test; chi-square test of independence; Fisher’s exact test. ^3^ OR, odds ratio; CI, confidence interval. ^4^ The unit of neighborhood unemployment is 1% proportion of population aged 16+ in the civilian labor force who are unemployed; the unit of neighborhood poverty is 1% proportion of population with annual income below the federal poverty level; and the unit of neighborhood education is 1% proportion of adults with less than high school diploma in 2010. The bold are indicate the significance.

**Table 4:** Sequences of testing results for PCR and serology tests of 3649 individuals who had at least one PCR test result preceding at least one serology test. Only test results before vaccination (or before 14 December 2020, when vaccination status is unknown) are included.

		Subsequent Serologic Test Results	
		Positive at Least Once	Always Negative
**Prior** **PCR Test Result**	Positive	522	371
	Negative	169	2587

**Table 5:** Vaccination status and timing.

	Vaccine		
	Pfizer-BioNTech	Moderna	Johnson&Johnson/Jannsen
*n*	5129	1304	161
Vaccination Status (%)			
Received 1 dose	1197 (23.3)	583 (44.7)	161 (100)
Received 2 doses	3932 (76.7)	721 (55.3)	n/a
Timing 2nd Vaccination (%)			
Early ^a^	4 (0.1)	3 (0.2)	n/a
Recommended ^b^	3778 (73.7)	697 (53.5)	n/a
Late/Allowable ^c^	140 (2.7)	20 (1.5)	n/a
Late ^d^	10 (0.2)	1 (0.1)	n/a
Pending, Late ^e^	228 (4.4)	309 (23.7)	n/a
Pending ^f^	969 (18.9)	274 (21.0)	n/a

^a^ Received second dose <17 days (Pfizer-BioNTech) or <24 days (Moderna) after first dose. ^b^ Received second dose 17–25 days (Pfizer-BioNTech) or 24–32 days (Moderna) after first dose. ^c^ Received second dose 26–42 days (Pfizer-BioNTech) or 33–42 days (Moderna) after first dose. ^d^ Received second dose >42 days after first dose. ^e^ No second dose received, >=42 days after first dose. ^f^ No second dose received, <42 days after first dose.

**Table 6:** Positivity rate among serology-tested individuals relative to the timing of the 1st and 2nd COVID vaccination doses. A total of 139 individuals were (temporarily) partially inoculated, while 347 individuals were fully inoculated with Moderna, Pfizer/BioNTech, or Johnson&Johnson/Janssen vaccines.

	Positive Tested Individuals Among Vaccinated (%)			
	Overall	Pfizer-BioNTech	Moderna	Johnson&Johnson/Janssen ^a^
*n*	472	355	103	14
Between 1st and 2nd Dose	78/139 (56.1)	52/98 (53.1)	26/41 (63.4)	n/a
Fully vaccinated ^b^	296/347 (85.3)	233/268 (86.9)	52/65 (80.0)	11/14 (78.6)
2–4 Weeks after Final Dose	125/153 (81.7)	99/119 (83.2)	21/27 (77.8)	5/7 (71.4)
4–8 Weeks after Final Dose	105/125 (84.0)	76/90 (84.4)	24/29 (82.8)	5/6 (83.3)
8–12 Weeks after Final Dose	55/60 (91.7)	47/50 (94.0)	7/9 (77.8)	1/1 (100.0)
>12 Weeks after Final Dose	19/21 (90.5)	18/20 (90.0)	1/1 (100.0)	n/a

Notes: Individuals who tested positive before vaccination and test results for “SARS-CoV-2 Total Antibody, Nucleocapsid” were excluded; some individuals were tested multiple times at various time points after the 1st dose. ^a^ Single dose vaccination. ^b^ at least 2 weeks after two doses of Pfizer-BioNTech or Moderna vaccines or at least 2 weeks after a single dose of Janssen vaccine.

## Data Availability

Data cannot be shared publicly due to patient confidentiality.

## Supplementary Materials

The following are available online at https://www.mdpi.com/article/10.3390/jcm10194341/s1. Figure S1: The purposes of four different tests: Tested versus random controls; tested positive versus tested negative and tested positive versus random controls, Figure S2: The composition of 10,416 individuals who had serologic tests, stratified by the date 14 December 2020, test results and vaccination information, Figure S3: Characteristics of 8026 individuals with at least a serologic test before 14 December 2020, stratified, stratified by the serologic results, Figure S4: Characteristics of 2024 individuals with at least a serologic test between 14 December 2020 and their first vaccination dose (Individuals who had positive serology test results before 14 December 2020 were excluded), stratified by the serologic results, Figure S5: Timing between 1st and 2nd vaccination of 4653 individuals who received 2 doses of Pfizer-BioNTech or Moderna vaccine, Figure S6: Distribution of time to first positive serologic result after the first and second dose, respectively, Table S1: Summary of test counts per month between 27 April 2020 and 3 May 2021, Table S2: Characteristics of 8026 individuals with at least a serologic test before 14 December 2020 and 24,078 unmatched controls, Table S3: Characteristics of 2024 individuals with at least a serologic test between 14 December 2020 and their first vaccination dose (Individuals who had positive serology test results before 14 December 2020 were excluded) and 6072 unmatched controls, Table S4: Sequences of testing results for PCR and serology tests of 8732 individuals who had at least one serology test.
