# Supplementary material for: Understanding the Patterns of Serological Testing for COVID-19 Pre- and Post-Vaccination Rollout in Michigan
Source: J Clin Med. 2021 Sep 24;10(19):4341. doi: 10.3390/jcm10194341 (PMC8509702; doi:10.3390/jcm10194341)

Supplementary Tables and Figures

Supplementary Table S1. Summary of Test Counts per Month between 27 April 2020 and 3 May 2021

Year	Month	# of Tests			# positive Tests	Monthly Positivity Rate
		Per Month	Daily Average	Daily Maximum		
2020	April*	29	7.3	11	13	45.7%
	May	332	10.7	42	91	25.9%
	June	1358	45.3	134	88	13.2%
	July	1492	48.1	116	88	6.6%
	August	2623	84.6	216	107	3.5%
	September	742	24.7	47	67	9.5%
	October	677	21.8	37	80	10.9%
	November	651	21.7	54	162	32.1%
	December	781	25.2	56	205	28.4%
2021	January	661	21.3	41	240	38.6%
	February	638	22.8	38	344	51.6%
	March	573	18.5	35	299	52.8%
	April	628	20.9	43	341	51.2%
	May**	42	14.0	33	19	46.1%

Note: The aggregated test counts include 295 tests (2.6%) that were repeated for the same person on the same day and had consistent results.

* last four days; **first three days

Supplementary Table S2. Characteristics of 8026 individuals with at least a serologic test before 14 December 2020 and 24,078 unmatched controls. Statistics presented are median (inter-quartile range) for continuous variables and *n* (%) for categorical variables. Unadjusted p-values are reported for either Wilcoxon rank-sum (continuous) or chi-square tests of independence (categorical) comparing the distributions of each of these characteristics between testing groups. Odds ratios and 95% confidence intervals are reported for each characteristic, fully adjusting for all other demographic and clinical characteristics in a logistic regression model. When fitting the logistic regression, the controls were randomly selected 20 times and the case control ratio was fixed as 1:3 each time. We finally pooled 20 estimates from each model into a single set of estimates.

	Unadjusted Comparisons			Adjusted Comparisons		
Characteristic	Unmatched controls, *n* = 24,078[1]	Individuals with at least a serologic test, *n* = 8,026[1]	*p*-value[2]	OR[3]	95% CI[3]	*p*-value
Age, per 10 years	4.30 (2.20, 6.40)	4.40 (2.80, 5.90)	**0.012**	0.84	0.82, 0.86	**<0.001**
Body Mass Index	27 (23, 32)	27 (23, 31)	**<0.001**	1.00	0.99, 1.00	0.60
Sex			**<0.001**			
Male	11,146 (46%)	3,100 (39%)		—	—	
Female	12,916 (54%)	4,926 (61%)		1.40	1.30, 1.51	**<0.001**
Race/Ethnicity			**<0.001**			
Non-Hispanic White	14,850 (62%)	6,116 (76%)		—	—	
Non-Hispanic Black	1,790 (7.4%)	543 (6.8%)		0.98	0.85, 1.14	0.80

	Unadjusted Comparisons			Adjusted Comparisons		
Characteristic	Unmatched controls, *n* = 24,078[1]	Individuals with at least a serologic test, *n* = 8,026[1]	*p*-value[2]	OR[3]	95% CI[3]	*p*-value
Other/Unknown	7,438 (31%)	1,367 (17%)		0.67	0.61, 0.74	**<0.001**
Smoking Status			**<0.001**			
Never	12,080 (69%)	5,427 (74%)		—	—	
Current/Former	5,313 (31%)	1,898 (26%)		0.74	0.69, 0.81	**<0.001**
Unknown	6,685 (28%)	701 (8.7%)		0.37	0.29, 0.47	**<0.001**
Neighborhood Unemployment[4]	6.3 (4.5, 8.5)	5.1 (3.8, 7.1)	**<0.001**	0.95	0.94, 0.97	**<0.001**
Neighborhood Poverty[4]	10 (5, 18)	6 (4, 12)	**<0.001**	0.98	0.98, 0.99	**<0.001**
Neighborhood Education[4]	7.4 (4.2, 12.3)	4.7 (2.7, 8.2)	**<0.001**	0.94	0.93, 0.95	**<0.001**
Population Density, 1000 persons per square mile	1.76 (0.38, 3.44)	1.84 (0.50, 3.43)	**<0.001**	1.06	1.04, 1.08	**<0.001**
Respiratory Diseases			**<0.001**			
No	13,324 (59%)	1,450 (20%)		—	—	
Yes	9,422 (41%)	5,920 (80%)		3.57	3.28, 3.88	**<0.001**
Circulatory Diseases			**<0.001**			
No	13,879 (61%)	2,518 (34%)		—	—	
Yes	8,867 (39%)	4,852 (66%)		1.91	1.76, 2.08	**<0.001**
Any Cancer			**<0.001**			
No	18,624 (82%)	5,673 (77%)		—	—	
Yes	4,122 (18%)	1,697 (23%)		1.11	1.01, 1.21	**0.024**
Type 2 Diabetes			**<0.001**			
No	20,971 (92%)	6,499 (88%)		—	—	
Yes	1,775 (7.8%)	871 (12%)		1.05	0.94, 1.18	0.40
Kidney Diseases			**<0.001**			
No	21,791 (96%)	6,811 (92%)		—	—	
Yes	955 (4.2%)	559 (7.6%)		0.98	0.85, 1.12	0.70
Liver Diseases			**<0.001**			
No	22,258 (98%)	6,776 (92%)		—	—	
Yes	488 (2.1%)	594 (8.1%)		2.10	1.80, 2.44	**<0.001**
Autoimmune Diseases			**<0.001**			
No	21,310 (94%)	6,122 (83%)		—	—	
Yes	1,436 (6.3%)	1,248 (17%)		1.66	1.49, 1.84	**<0.001**

[1]Statistics presented: median (IQR); *n* (%).
[2]Statistical tests performed: Wilcoxon rank-sum test; chi-square test of independence; Fisher's exact test.
[3]OR = Odds Ratio, CI = Confidence Interval
[4]The unit of neighborhood unemployment is 1% proportion of population age 16+ in the civilian labor force who are unemployed; the unit of neighborhood poverty is 1% proportion of population with annual income below the federal poverty level; and the unit of neighborhood education is 1% proportion of adults with less than high school diploma in 2010.

Supplementary Table S3. Characteristics of 2,024 individuals with at least a serologic test between December 14, 2020 and their first vaccination dose (Individuals who had positive serology test results before December 14, 2020 were excluded) and 6,072 unmatched controls. Statistics presented are median (inter-quartile range) for continuous variables and *n* (%) for categorical variables. Unadjusted p-values are reported for either Wilcoxon rank-sum (continuous) or chi-square tests of independence (categorical) comparing the distributions of each of these characteristics between testing groups. Odds ratios and 95% confidence intervals are reported for each characteristic, fully adjusting for all other demographic and clinical characteristics in a logistic regression model. When fitting the logistic regression, the controls were randomly selected 20 times and the case control ratio was fixed as 1:3 each time. We finally pooled 20 estimates from each model into a single set of estimates.

	Unadjusted Comparisons			Adjusted Comparisons		
Characteristic	Unmatched controls, *n* = 6,072[1]	Individuals with at least a serologic test, *n* = 2,024[1]	*p*-value[2]	OR[3]	95% CI[3]	*p*-value
Age, per 10 years	4.40 (2.30, 6.50)	4.20 (2.20, 5.80)	**<0.001**	0.84	0.79,0.89	**<0.001**
Body Mass Index	27 (24, 32)	27 (23, 31)	**0.013**	0.99	0.98,1.01	0.388
Sex			**<0.001**			
Male	2,809 (46%)	807 (40%)		—	—	
Female	3,261 (54%)	1,217 (60%)		1.32	1.09,1.59	**0.005**
Race/Ethnicity			**<0.001**			
Non-Hispanic White	3,752 (62%)	1,541 (76%)		—	—	
Non-Hispanic Black	436 (7.2%)	113 (5.6%)		0.88	0.62,1.23	0.447
Other/Unknown	1,884 (31%)	370 (18%)		0.84	0.65,1.07	0.153
Smoking Status			**0.016**			
Never	3,008 (69%)	1,362 (72%)		—	—	
Current/Former	1,326 (31%)	518 (28%)		0.84	0.7,1	0.055
Unknown	1,738 (29%)	144 (7.1%)		0.14	0.06,0.31	**<0.001**
Neighborhood Unemployment[4]	6.3 (4.6, 8.5)	5.3 (3.9, 7.2)	**<0.001**	0.95	0.92,0.99	**0.007**
Neighborhood Poverty[4]	10 (5, 18)	7 (4, 14)	**<0.001**	0.99	0.98,1	0.175
Neighborhood Education[4]	7.5 (4.2, 12.3)	5.0 (2.9, 8.7)	**<0.001**	0.96	0.94,0.98	**<0.001**
Population Density, 1000 persons per square mile	1.78 (0.38, 3.49)	1.53 (0.43, 3.24)	0.3	1.01	0.97,1.06	0.524
Respiratory Diseases			**<0.001**			
No	3,363 (59%)	355 (19%)		—	—	
Yes	2,363 (41%)	1,497 (81%)		3.84	3.12,4.71	**<0.001**
Circulatory Diseases			**<0.001**			
No	3,489 (61%)	640 (35%)		—	—	
Yes	2,237 (39%)	1,212 (65%)		2.16	1.74,2.68	**<0.001**
Any Cancer			**<0.001**			
No	4,672 (82%)	1,408 (76%)		—	—	
Yes	1,054 (18%)	444 (24%)		1.26	1.03,1.56	**0.028**
Type 2 Diabetes			**<0.001**			
No	5,287 (92%)	1,613 (87%)		—	—	
Yes	439 (7.7%)	239 (13%)		1.14	0.85,1.52	0.396
Kidney Diseases			**<0.001**			
No	5,484 (96%)	1,674 (90%)		—	—	
Yes	242 (4.2%)	178 (9.6%)		1.35	0.98,1.86	0.069
Liver Diseases			**<0.001**			
No	5,604 (98%)	1,714 (93%)		—	—	
Yes	122 (2.1%)	138 (7.5%)		1.89	1.35,2.63	**<0.001**
Autoimmune Diseases			**<0.001**			
No	5,360 (94%)	1,479 (80%)		—	—	
Yes	366 (6.4%)	373 (20%)		2.22	1.73,2.87	**<0.001**

[1]Statistics presented: median (IQR); *n* (%).
[2]Statistical tests performed: Wilcoxon rank-sum test; chi-square test of independence; Fisher's exact test.
[3]OR = Odds Ratio, CI = Confidence Interval
[4]The unit of neighborhood unemployment is 1% proportion of population age 16+ in the civilian labor force who are unemployed; the unit of neighborhood poverty is 1% proportion of population with annual income below the federal poverty level; and the unit of neighborhood education is 1% proportion of adults with less than high school diploma in 2010.

Supplementary Table S4. Sequences of testing results for PCR and serology tests of 8,732 individuals who had at least one serology test. Only test results before vaccination (or before December 14, 2020, when vaccination status is unknown) are included.

**Test Results[1]**	***n***	**Percent**
1 Test, n = 3,623 individuals (41.49%)		
O	3,403	93.9
X	220	6.1
2 Tests, n = 4,875 individuals (55.83%)		
-o	2490	51.1
o-	1093	22.4
+x	465	9.5
+o	332	6.8
o+	246	5
-x	151	3.1
x+	34	0.7
x-	27	0.6
Oo	26	0.5
Xx	6	0.1
Ox	4	0.1
Xo	1	0.02
3 Tests, n = 216 individuals (2.47%)		
-oo	82	38
+xx	35	16.2
+oo	18	8.3
o+o	17	7.9
o-o	12	5.6
o+x	11	5.1
-xx	10	4.6
oo-	8	3.7
+ox	6	2.8
-ox	5	2.3
oo+	4	1.9
x+x	3	1.4
-xo	1	0.5
ox+	1	0.5
xx+	1	0.5
ooo	1	0.5
oxo	1	0.5
4 Tests, n = 14 individuals (0.16%)		
+xxx	5	35.7
-ooo	2	14.3
o+xx	2	14.3
-oxx	1	7.1
-xxx	1	7.1
o+oo	1	7.1
oo+o	1	7.1
ooo+	1	7.1
5 Tests, n = 3 individuals (0.03%)		
+oooo	1	33.3
+xxxx	1	33.3
xoox+	1	33.3
7 Tests, n = 1 individual (0.01%)		
+oooooo	1	100

Supplementary Figure S1. The purposes of four different tests: Tested versus random controls; tested positive versus tested negative and tested positive versus random controls.

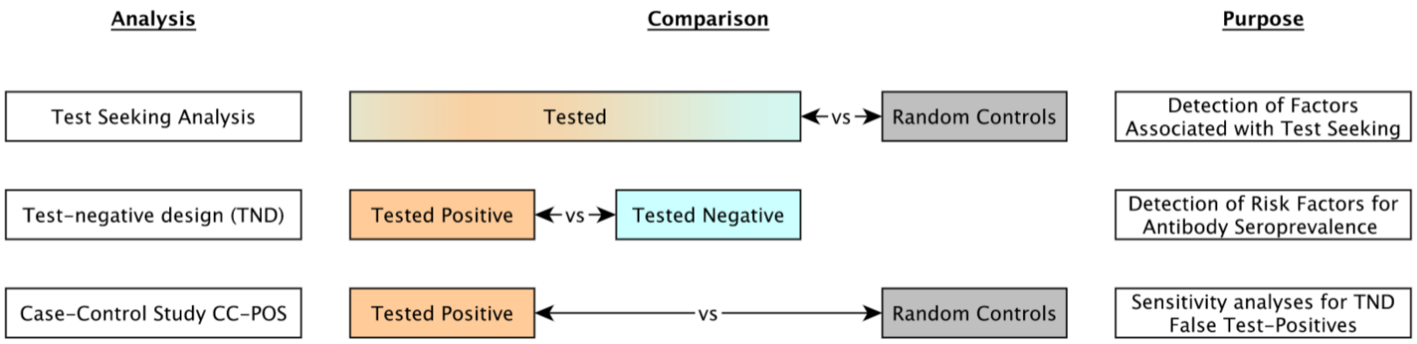

Supplementary Figure S2. The composition of 10,416 individuals who had serologic tests, stratified by the date 14 December 2020, test results and vaccination information.

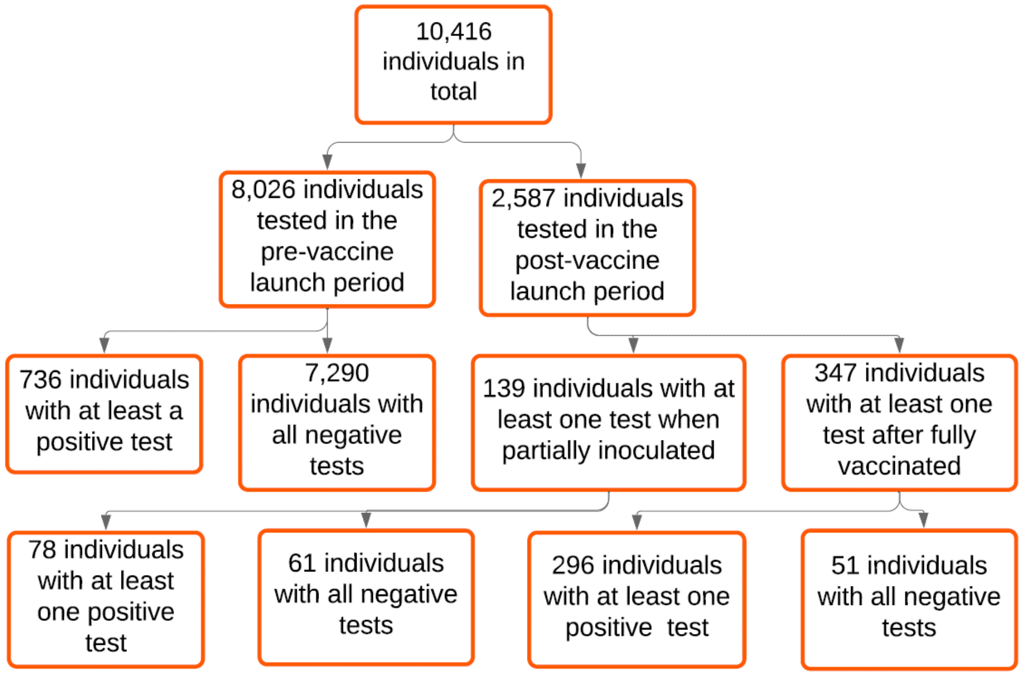

Supplementary Figure S3. Characteristics of 8026 individuals with at least a serologic test before 14 December 2020, stratified, stratified by the serologic results. 736 individuals had at least one positive test while 7290 individuals had all negative tests. Odds ratios and 95% confidence intervals are reported for each characteristic, fully adjusting for all other demographic and clinical characteristics in a logistic regression model. When fitting the logistic regression, the controls were randomly selected 20 times and the case control ratio was fixed as 1:3 each time. We finally pooled 20 estimates from each model into a single set of estimates.

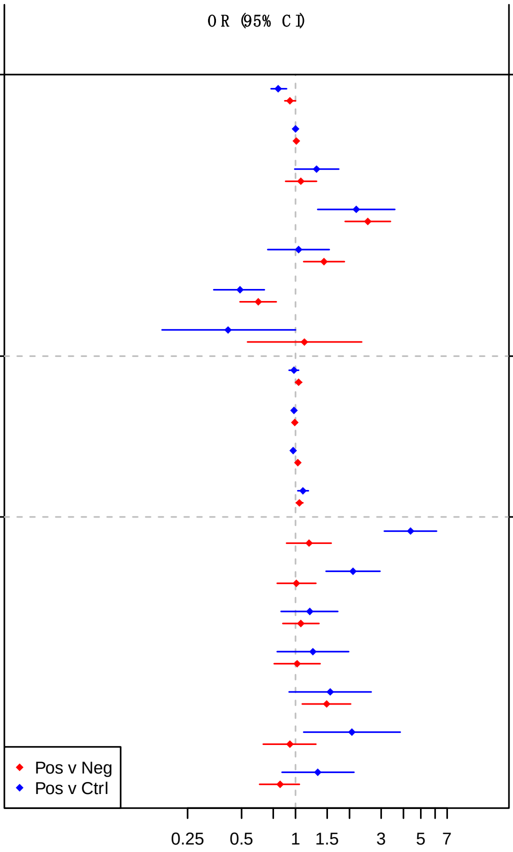

	Predictor	Reference	Pos v Neg OR	Pos v Neg 95% CI (L	U)	Pos v Ctrl OR	Pos v Ctrl 95% CI (L	U)	Pos v Ctrl P
Demographics	Age (10-year)		0.93	0.87	1.00	0.80	0.73	0.89	<0.001
	Body Mass Index		1.01	0.99	1.02	1.00	0.98	1.03	0.668
	Female	Male	1.07	0.88	1.31	1.31	0.99	1.74	0.061
	NH-Black	NH-White	2.53	1.89	3.38	2.18	1.33	3.58	0.003
	Other	NH-White	1.44	1.11	1.87	1.04	0.70	1.54	0.855
	Ever-Smoker	Never-Smoker	0.62	0.49	0.78	0.49	0.35	0.67	<0.001
	Smoking Unknown	Never-Smoker	1.12	0.54	2.34	0.42	0.18	1.00	0.052
SES	Unemployment		1.04	1.00	1.08	0.98	0.92	1.04	0.533
	Poverty		0.99	0.98	1.01	0.98	0.97	1.00	0.052
	Education		1.03	1.00	1.05	0.97	0.94	1.00	0.085
	Population Density (1k/mile^2)		1.05	1.01	1.10	1.10	1.03	1.18	0.007
Comorbidities	Respiratory Diseases		1.19	0.89	1.58	4.38	3.13	6.12	<0.001
	Circulatory Diseases		1.01	0.79	1.30	2.09	1.48	2.96	<0.001
	Any Cancer		1.07	0.85	1.35	1.20	0.83	1.72	0.328
	Type 2 Diabetes		1.02	0.76	1.37	1.25	0.79	1.98	0.349
	Kidney Diseases		1.49	1.09	2.03	1.56	0.92	2.64	0.10
	Liver Diseases		0.93	0.66	1.30	2.06	1.11	3.84	0.024
	Autoimmune Diseases		0.82	0.63	1.05	1.33	0.84	2.12	0.227

Note: The unit of neighborhood unemployment is 1% proportion of population age 16+ in the civilian labor force who are unemployed; the unit of neighborhood poverty is 1% proportion of population with annual income below the federal poverty level; and the unit of neighborhood education is 1% proportion of adults with less than high school diploma in 2010.

Supplementary Figure S4. Characteristics of 2024 individuals with at least a serologic test between 14 December 2020 and their first vaccination dose (Individuals who had positive serology test results before 14 December 2020 were excluded), stratified by the serologic results. 733 individuals had at least one positive test while 1291 individuals had all negative tests. Odds ratios and 95% confidence intervals are reported for each characteristic, fully adjusting for all other demographic and clinical characteristics in a logistic regression model. When fitting the logistic regression, the controls were randomly selected 20 times and the case control ratio was fixed as 1:3 each time. We finally pooled 20 estimates from each model into a single set of estimates.

	Predictor	Reference	Pos v Neg OR	Pos v Neg 95% CI (L)	Pos v Neg 95% CI (U)	Pos v Ctrl OR	Pos v Ctrl 95% CI (L)	Pos v Ctrl 95% CI (U)	Pos v Ctrl P
Demographics	Age (10-year)		0.97	0.89	1.05	0.82	0.75	0.91	<0.001
	Body Mass Index		1.02	1.00	1.04	1.00	0.98	1.03	0.678
	Female	Male	0.86	0.68	1.10	1.20	0.90	1.61	0.215
	NH−Black	NH−White	1.03	0.63	1.69	0.92	0.50	1.69	0.787
	Other	NH−White	1.20	0.86	1.67	0.88	0.59	1.31	0.526
	Ever−Smoker	Never−Smoker	0.84	0.65	1.09	0.70	0.52	0.96	0.025
	Smoking Unknown	Never−Smoker	2.62	0.54	12.7	0.22	0.07	0.71	0.012
SES	Unemployment		1.08	1.02	1.13	1.00	0.94	1.06	1
	Poverty		0.99	0.97	1.00	0.98	0.96	1.00	0.06
	Education		1.00	0.97	1.02	0.96	0.93	0.99	0.018
	Population Density (1k/mile^2)		1.01	0.95	1.08	1.02	0.95	1.10	0.599
Comorbidities	Respiratory Diseases		0.75	0.55	1.04	3.09	2.24	4.26	<0.001
	Circulatory Diseases		0.84	0.61	1.15	2.02	1.44	2.84	<0.001
	Any Cancer		0.99	0.75	1.31	1.21	0.85	1.72	0.288
	Type 2 Diabetes		0.99	0.69	1.41	1.23	0.78	1.94	0.383
	Kidney Diseases		1.18	0.80	1.74	1.64	0.98	2.73	0.059
	Liver Diseases		0.99	0.65	1.48	2.05	1.08	3.89	0.029
	Autoimmune Diseases		1.08	0.82	1.42	2.53	1.61	3.96	<0.001

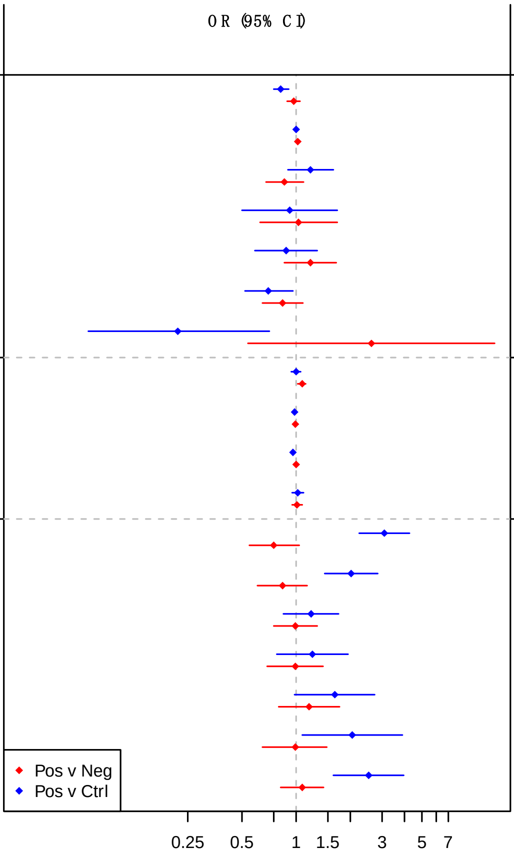

Note: The unit of neighborhood unemployment is 1% proportion of population age 16+ in the civilian labor force who are unemployed; the unit of neighborhood poverty is 1% proportion of population with annual income below the federal poverty level; and the unit of neighborhood education is 1% proportion of adults with less than high school diploma in 2010.

Supplementary Figure S5. Timing between 1st and 2nd vaccination of 4,653 individuals who received 2 doses of Pfizer-BioNTech or Moderna vaccine. Recommendation for second dose 17–25 days (Pfizer-BioNTech) or 24–32 days (Moderna) after first dose. Allowable timing for second dose 26–42 days (Pfizer-BioNTech) or 33–42 days (Moderna) after first dose.

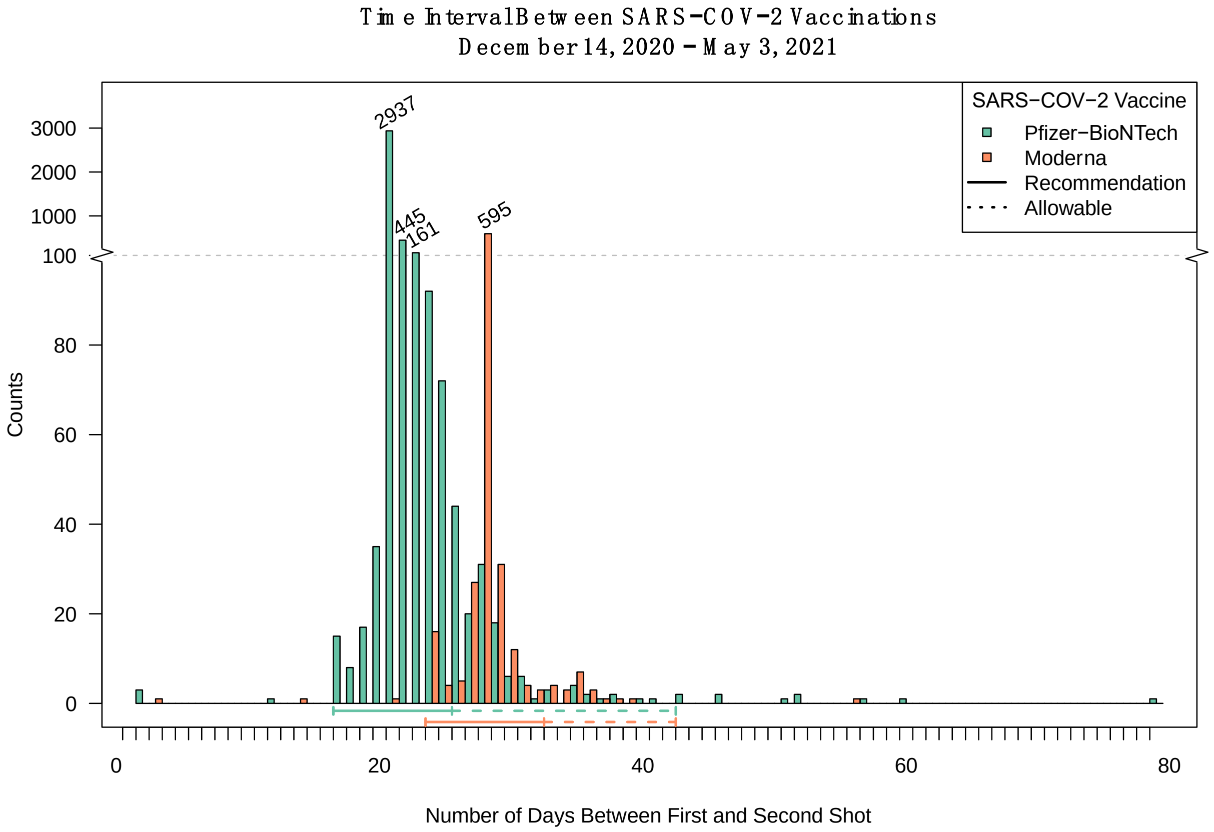

Supplementary Figure S6. Distribution of time to first positive serologic result after the first and second dose, respectively. Individuals who tested positive before vaccination were excluded and serology test results for "SARS-CoV-2 Total Antibody, Nucleocapsid" were excluded. The red line shows the value of median.

(a) Distribution of time to first positive serologic result between the first and second vaccination. (median: 21 days;1st quantile: 16.3 days; mean: 25.0 days; 3rd quantile: 30.5 days)

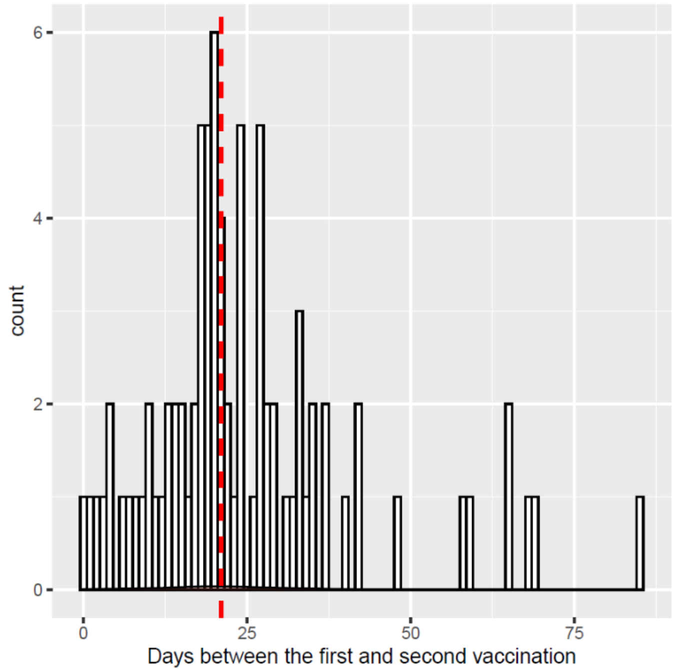

(b) Distribution of time to first positive serologic result after the second vaccination (median: 27.0 days; 1st quantile: 17.0 days; mean: 34.1 days; 3rd quantile: 45.0 days)

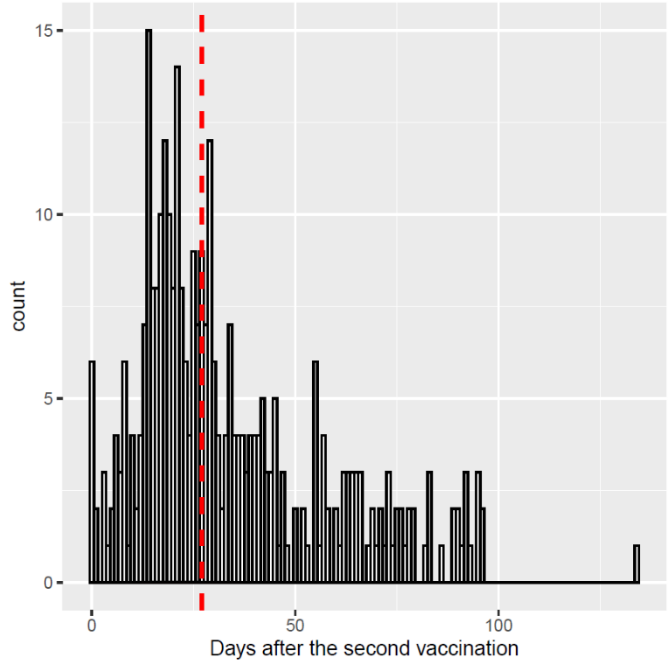

Supplement: Supplementary file 1 [file jcm-10-04341-s001.zip › jcm-1339247-supplementary.pdf]
